# Rational Design of a New Class of Toll-Like Receptor 4 (TLR4) Tryptamine Related Agonists by Means of the Structure- and Ligand-Based Virtual Screening for Vaccine Adjuvant Discovery

**DOI:** 10.3390/molecules23010102

**Published:** 2018-01-04

**Authors:** Jan Honegr, Rafael Dolezal, David Malinak, Marketa Benkova, Ondrej Soukup, Joyce S. F. D. de Almeida, Tanos C. C. Franca, Kamil Kuca, Roman Prymula

**Affiliations:** 1 Biomedical Research Centre, University Hospital Hradec Kralove, Sokolska 581, 50005 Hradec Kralove, Czech Republic; jan.honegr@gmail.com (J.H.); rafael.dolezal@fnhk.cz (R.D.); david.malinak@gmail.com (D.M.); marketa.pasdiorova@fnhk.cz (M.B.); ondrej.soukup@fnhk.cz (O.S.); kamil.kuca@fnhk.cz (K.K.); 2 Department of Forensic Medicine and Intensive Medicine, Faculty of Medicine, University of Ostrava, Syllabova 19, Ostrava 70300, Czech Republic; 3 Department of Chemistry, Faculty of Science, University of Hradec Kralove, Rokitanskeho 62, 50003 Hradec Kralove, Czech Republic; 4 Center for Basic and Applied Research, Faculty of Informatics and Management, University of Hradec Kralove, Rokitanskeho 62, 50003 Hradec Kralove, Czech Republic; tanosfranca@gmail.com; 5 Laboratory of Molecular Modeling Applied to Chemical and Biological Defense, Military Institute of Engineering, Praca General Tiburcio 80, Rio de Janeiro 22290-270, Brazil; joycesfdalmeida@gmail.com

**Keywords:** PRR, TLR4, innate immunity, virtual screening, molecular dynamics, adjuvants

## Abstract

In order to identify novel lead structures for human toll-like receptor 4 (*h*TLR4) modulation virtual high throughput screening by a peta-flops-scale supercomputer has been performed. Based on the in silico studies, a series of 12 compounds related to tryptamine was rationally designed to retain suitable molecular geometry for interaction with the *h*TLR4 binding site as well as to satisfy general principles of drug-likeness. The proposed compounds were synthesized, and tested by in vitro and ex vivo experiments, which revealed that several of them are capable to stimulate *h*TLR4 in vitro up to 25% activity of Monophosphoryl lipid A. The specific affinity of the in vitro most potent substance was confirmed by surface plasmon resonance direct-binding experiments. Moreover, two compounds from the series show also significant ability to elicit production of interleukin 6.

## 1. Introduction

In recent years, we are experiencing outbreaks of epidemics of diseases like measles or rubeola against which we already have preventive vaccines [1]. It must be said that such outbreaks occur mainly in the so called “Western world”, and are caused by anti-systemic paranoia, conspiratorial theorists or just individuals who want to make their profit out of uninformed people. One of the biggest issues with vaccination is mainly their administration in small children or even newborns, which makes parents extremely sensitive when giving anything potentially harmful to their beloved ones [2].

One of the core beliefs, besides that vaccines causes autism, is that vaccines contain dangerous adjuvants like mercury, aluminium, squalene, animal cells, formaldehyde and many other suspicious substances [3]. Such opinions are partially true because preparation of actual vaccines is a multi-step process requiring various agents and tools. It is undeniable that most vaccines contain alum, or that thiomersal is used in the early stages of the manufacturing process of some vaccines, or directly added to anti-influenza vaccines (e.g., Pandermix) [4]. On the other hand, it has been proved beyond any doubt by a vast number of studies [5] that mercury or aluminium in vaccines do not cause autism or behavioral problems like attention deficit disorder (ADHD). Despite this fact, claims that vaccines are generally not safe sometimes come from unexpected, but highly influential sources like the Court of Justice of the European Union [6]. This is the reason why development of novel vaccines containing safer adjuvants is necessary.

Other factors complicating the willingness for application of vaccines are a worldwide focus on the health safety, and limitation of their adverse effect potential. This leads to the development of novel types of subunit, or DNA vaccines, which are much safer than traditional attenuated or killed vaccines, but in the same time they are not as effective in eliciting strong and long-lasting protection [7]. This is caused by lack of naturally adjuvanting compounds that innate immunity system recognize as danger associated molecular patterns (DAMPs). The most potent ones is the endotoxin, also known as lipopolysaccharide (LPS), constituted by the polymeric complex moiety which is found in the outer membrane of Gram-negative bacteria, and which is responsible for eliciting very strong immune responses in animals [8].

LPS is recognized in organisms by divergent biochemical pathways. One pathway uses natural antibodies and lipoproteins to neutralize and clear LPS. The second pathway triggers a vigorous and complex inflammatory response involving activation of immunocompetent cells (such as monocytes, macrophages, and hepatic Kupffer cells) through lipopolysaccharide binding protein (LBP), cell surface-bound protein coded by the cluster of differentiation 14 gene (CD14), and toll-like receptor 4 (TLR4). TLR4 activation by bacterial LPS is achieved by the coordinate and sequential action of three other proteins, the lipopolysaccharide binding protein (LBP), the cluster differentiation antigen CD14, and the myeloid differentiation protein (MD2) receptors. The three receptors bind the LPS and present it in a monomeric form to the TLR4 by forming the activated dimer (*h*TRL4/MD2-LPS)_2_ complex. Identification of LPS triggers the complex regulatory cascade, leading to the production of pro-inflammatory cytokines (IL-1, TNF-α, IL-6, IL-8 etc.) that can protect the host. [9]

LPS is a highly toxic substance causing septic shock and septicaemia, thus, efforts in diminishing of its toxic properties have been made. It was found that the part mainly responsible for the immunogenic properties of LPS is the so-called Lipid A. Later, a derivative of Lipid A—Monophosphoryl lipid A (MPLA) has been introduced as a vaccine adjuvant. However, the complexity of MPLA urges rational design of simpler compounds that retain intrinsic activity on *h*TRL4/MD2 and are easier to prepare and handle [9,10].

**Figure 1:**
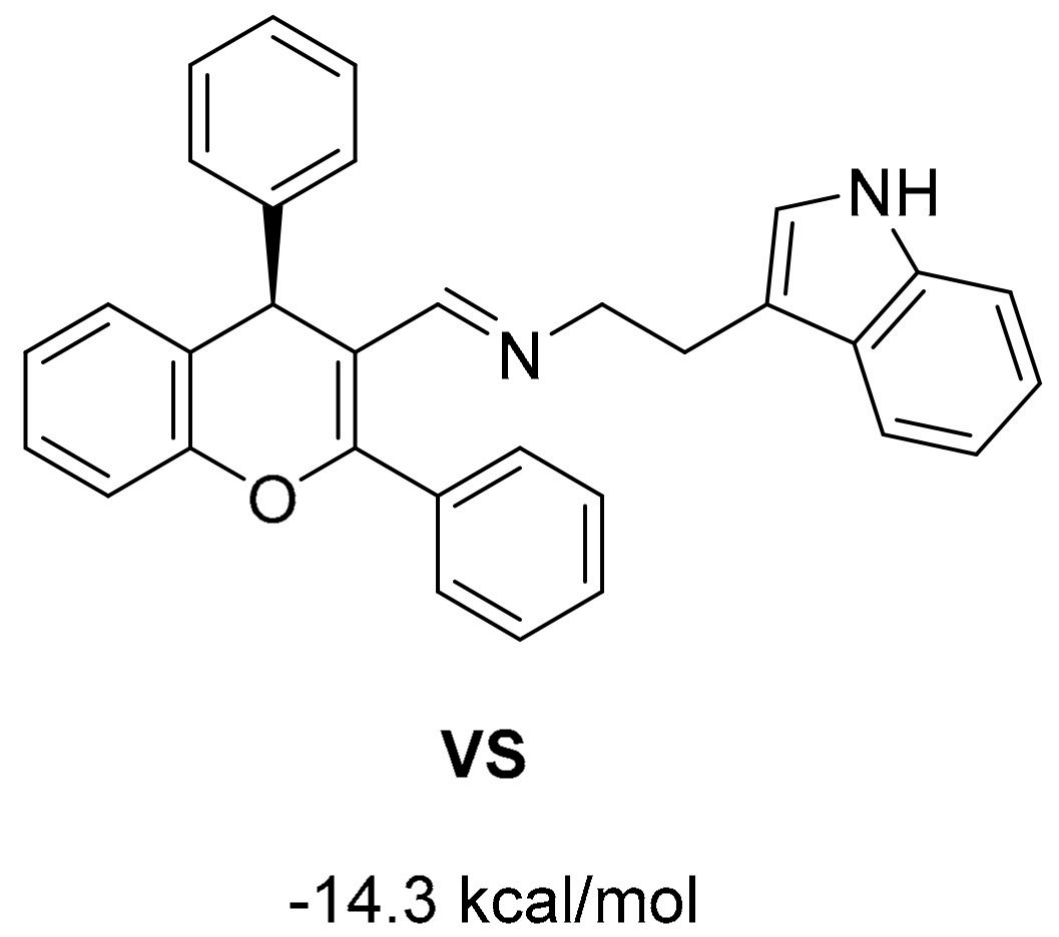
A hit structure **VS** selected by virtual high throughput screening and its predicted binding energy for *h*TRL4/MD2 (PDB ID: 4G8A).

In the present study we focused on rational design of novel *h*TRL4/MD2 modulators employing virtual high throughput screening (vHTPS) with the use of a peta-flops-scale super computer [11]. From several promising substances identified in this in silico study, we have selected a chemical structure (**VS**) which exhibited considerable in silico binding affinity towards *h*TRL4/MD2 model (i.e., binding energy estimate of −14.3 kcal/mol) (Figure 1). This compound can be found in the online ZINC database under code ZINC02157367.

**Figure 2:**
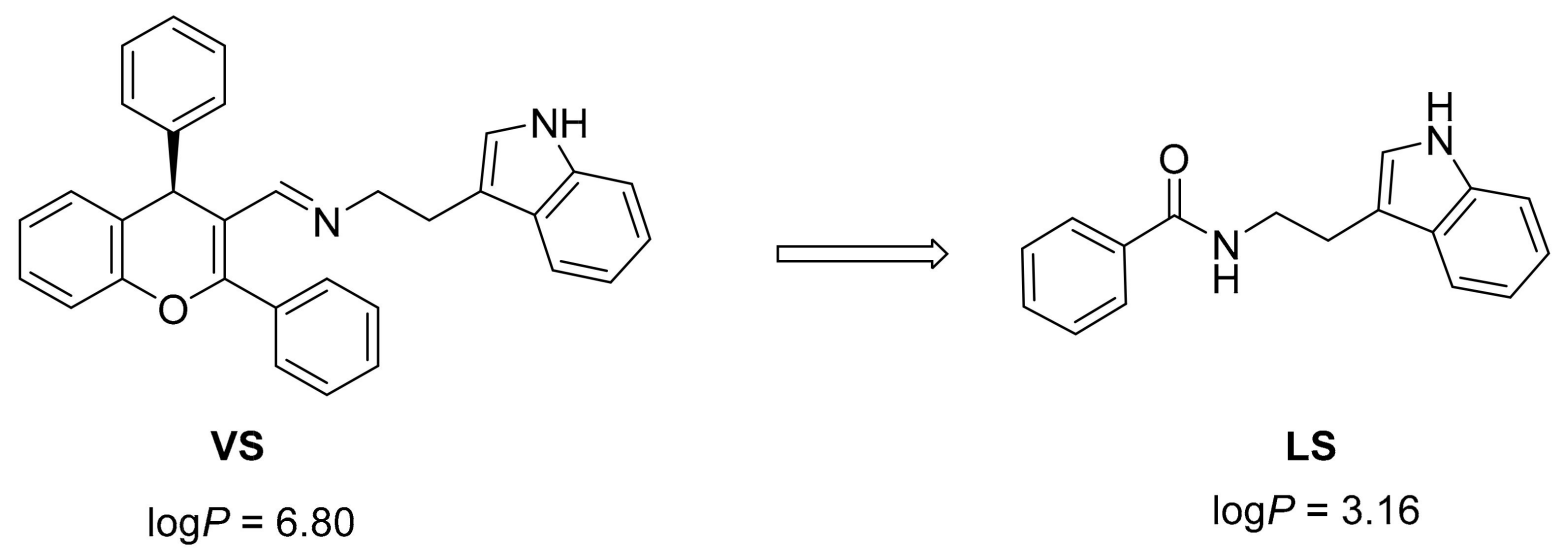
Optimization of the **VS** hit structure identified by virtual screening to less lipophilic lead structure **LS**. The log*P* values were calculated in MarvinSketch 15.12.7.0.

In order to optimize drug-like properties of the hit structure **VS** indicated by virtual screening and to keep, to certain extent, its main structural features responsible for the interactions with *h*TRL4/MD2 in the same time, a novel structure **LS** was designed with the aim to achieve especially a lower lipophilicity (Figure 2).

Therefore, the first objective of the present work was to design 12 novel structures related to *N*-(2-(1*H*-indol-3-yl)ethyl)benzamide (**LS**), to predict their affinity for *h*TRL4/MD2 by flexible molecular docking and molecular dynamics. At second, the practical part of the work involved synthesis and testing of the compounds by in vitro and ex vivo experiments to confirm the predicted properties.

## 2. Results and Discussion

### 2.1. Virtual Screening and Rational Design

**Figure 3:**
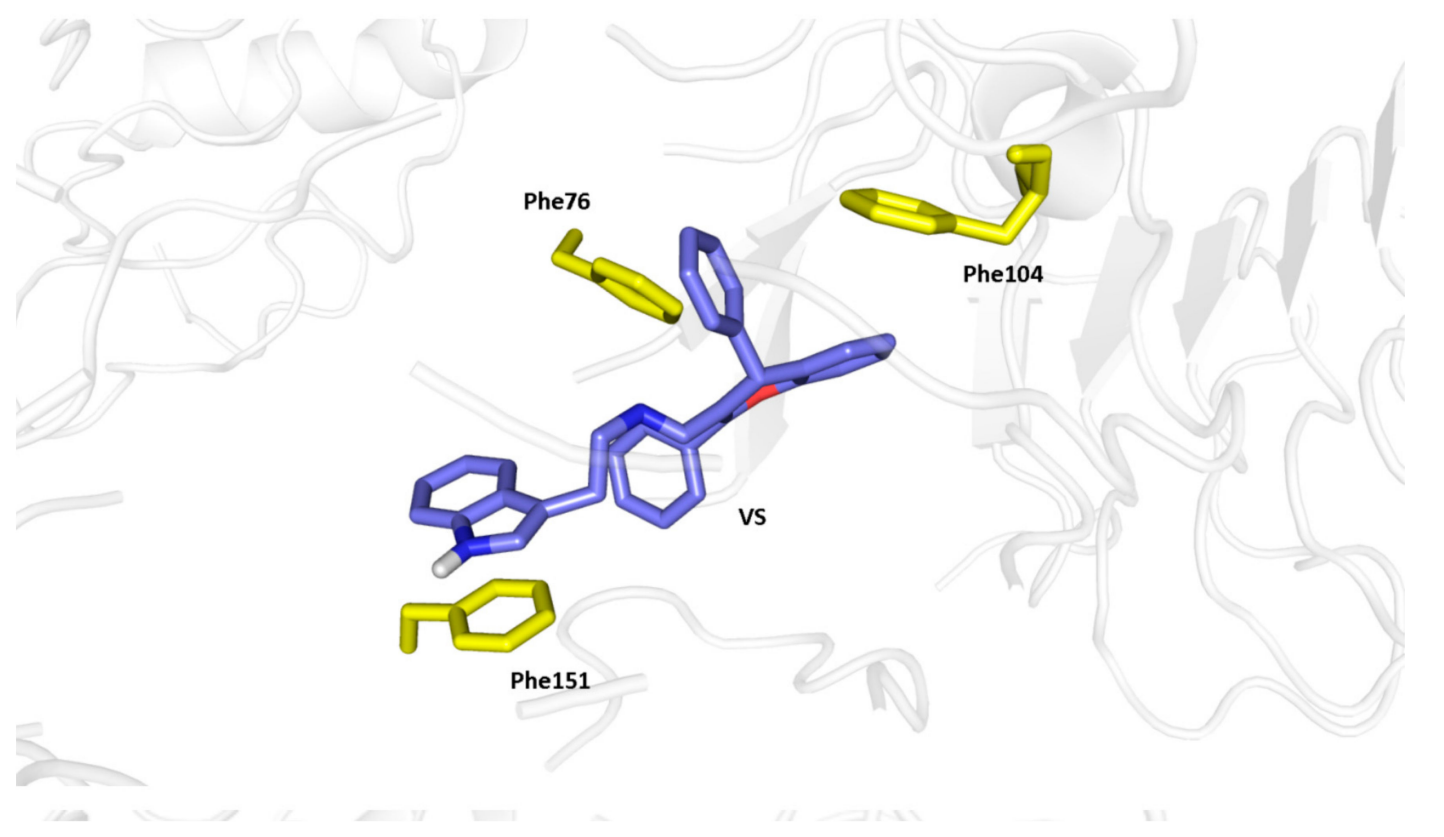
Binding mode of **VS** in *h*TRL4/MD2 (PDB ID: 4G8A) determined by structure-based virtual screening. The carbon atoms of the ligand are colored in blue, the aminoacid residues are colored in yellow.

In this study, we have performed a combined ligand and structure based virtual screening of 130,000 compounds using a peta-flops-scale supercomputer to find novel potential modulators of *h*TRL4/MD2 (PDB ID: 4G8A). This hierarchical in silico methodology consisted in a ligand based virtual screening of a random virtual library containing 130,000 structures from the ZINC database (http://zinc.docking.org/), MW ≤ 500, xlogp ≤ 7, HBD ≤ 5, and HBA ≤ 10. Using known *h*TRL4/MD2 agonists and antagonists as templates (e.g., carbamazepine, fentanyl, morphine, loverphaol, ibudilast, naloxone, etc.), 9700 structures were selected from the starting database, having a Tanimoto similarity index with respect to the template structures higher than 0.7. Another 300 structures were chosen from our in-house compound library and from the literature [10,12,13,14,15,16,17]. In total, 10,000 structures were submitted to flexible molecular docking in AutoDock Vina 1.1.2. to estimate their binding energy in *h*TRL4/MD2 (PDB ID: 4G8A). Since the computational demands for the structure-based virtual screening required roughly 500,000 core-hours, the Anselm supercomputer was used (IT4Innovations, National Supercomputing Center, http://www.it4i.cz). From the top scoring candidates, we selected the structure **VS**, which exhibited very promising estimate of the binding energy in *h*TRL4/MD2. Taking into account that the vast majority of biochemical experiments needs water soluble substances, an intuitive design of **VS** was made to achieve a structure with a lower lipophilicity. Investigating the predicted binding mode of **VS**, we simplified the hit structure to *N*-(2-(1*H*-indol-3-yl)ethyl)benzamide (**LS**, Figure 2), which retains the tryptamine moiety to interact with Phe151 in the active site of *h*TRL4/MD2 (PDB ID: 4G8A) and is capable to bind by its phenyl ring with Phe76 or Phe104 (Figure 3).

These structural requirements for ligands to bind in mostly hydrophobic active site of *h*TRL4/MD2 were considered together along with general principles of rational drug design, which recommend to follow for instance the classical Lipinski’s rule of five. The structure **LS** was proposed to exhibit sufficient affinity towards *h*TRL4/MD2 as well to be more water soluble and easily synthesizable. Based on these ideas, 12 different compounds related to **LS** were designed, analyzed by flexible molecular docking in *h*TRL4/MD2, synthesized and tested by in vitro and ex vivo experiments. Finally, the observed in vitro activities of the synthesized compounds in *h*TRL4/MD2 were compared with the results of molecular dynamic simulations which provided optimized binding energy estimates by Poisson-Boltzmann surface area continuum solvation method (MM/PBSA).

### 2.2. Chemistry

**Scheme 1:**
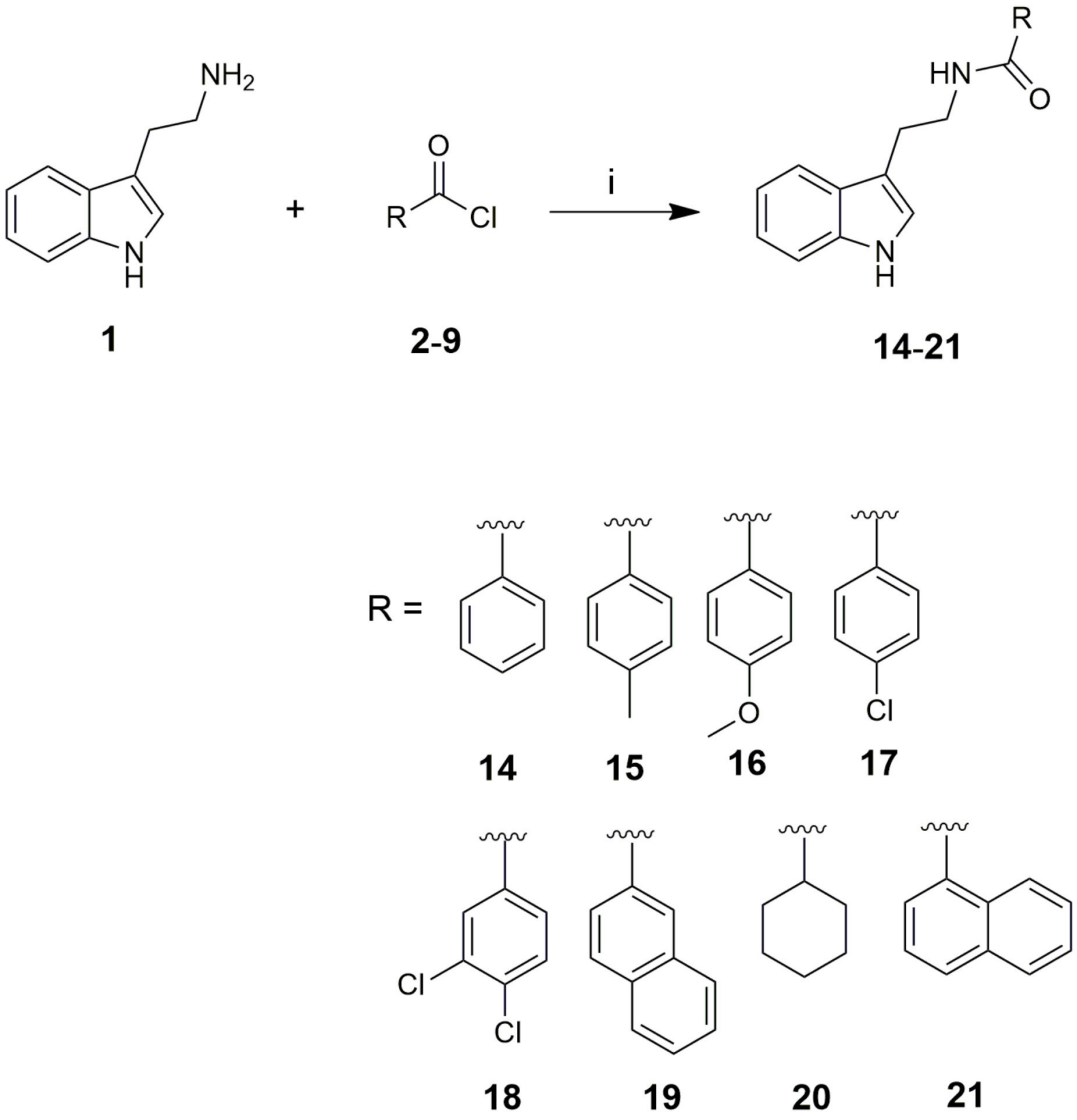
Synthesis of compounds **14**–**21** (Procedure A). *Reagents and conditions*: (i) Et_3_N, CH_2_Cl_2_, 0 °C→r.t., 1 h, 59–92%.

The compounds **14**–**25** were synthesized by two different procedures (A and B). In this work both previously synthesized tryptamine derivatives **14**–**20** [18,19,20,21,22,23,24,25] and new tryptamine derivatives **21**–**25** have been prepared. Products **14**–**21** were prepared by nucleophilic acyl substitution of tryptamine (**1**) by the commercially available acyl chlorides **2**–**9**. The reactions were carried out in CH_2_Cl_2_ in the presence of Et_3_N as the base. Reaction yields were in the range 59–92% (Scheme 1) (Procedure A).

**Scheme 2:**
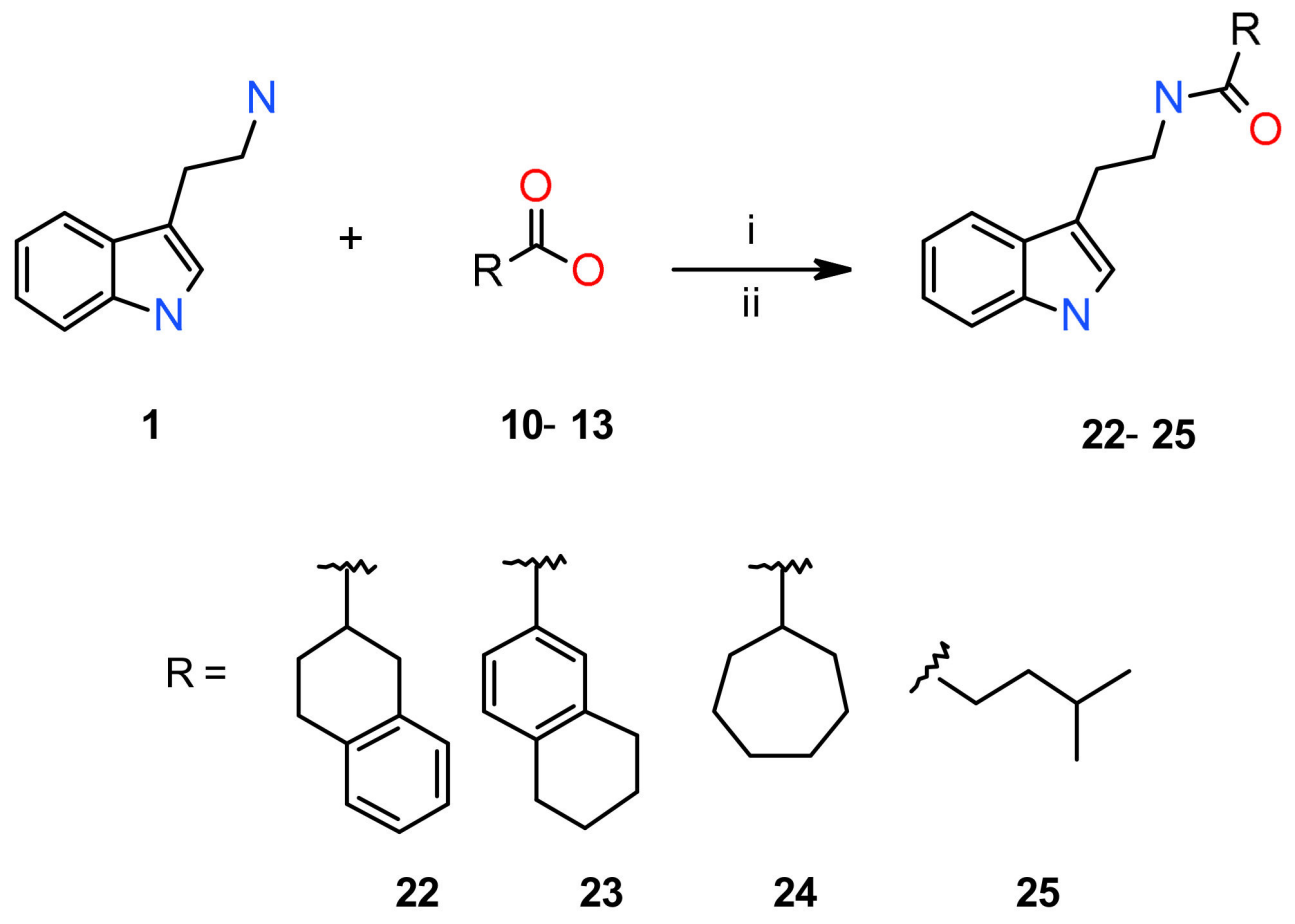
Synthesis of compounds **22**–**25** (Procedure B). *Reagents and conditions*: (i) (COCl)_2_, DMF, carboxylic acids **10**–**13**, CH_2_Cl_2_, 0 °C→r.t., 1 h; (ii) Et_3_N, CH_2_Cl_2_, 0 °C→r.t., 1 h, 81–91%.

In the procedure B, the appropriate acyl chlorides were first prepared in situ by reaction of commercially available carboxylic acids **10**–**13** with (COCl)_2_ and a catalytic amount of DMF in CH_2_Cl_2_. Subsequently, nucleophilic acyl substitution of compound **1** by the prepared acyl chlorides in CH_2_Cl_2_ in the presence of Et_3_N as the base provided final products **22**–**25** in 81–91% yields after two steps (Scheme 2). The course of all reactions was monitored by TLC, and the structures of all products after column chromatography purification were determined by ^1^H-, ^13^C-NMR and HRMS analysis. The melting points were also determined for the characterization of the synthesized compounds **14**–**25** (Table 1). Based on LC with UV detection (*λ* = 254 nm), the non-calibrated purity of final products was ≥95%.

### 2.3. Molecular Docking Study

**Figure 4:**
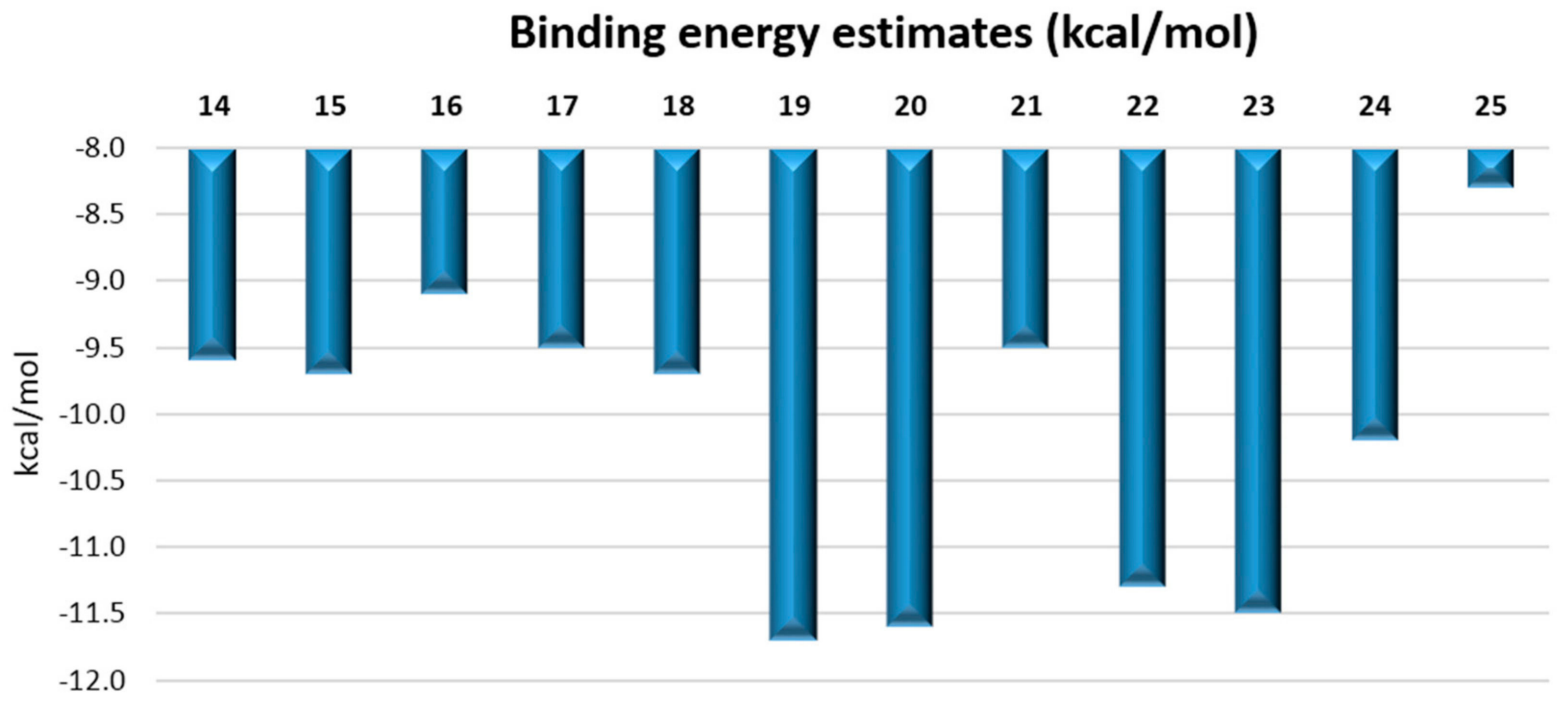
Binding energy estimates of **14**–**25** for *h*TRL4/MD2-LPS (PDB ID: 4G8A) determined by flexible molecular docking.

The interactions of **14**–**25** compounds with *h*TRL4/MD2 (PDB ID: 4G8A) were simulated by flexible molecular docking using AutoDock Vina 1.1.2 software [26]. The active site for the in silico study was determined by the position of LPS co-crystalized with the receptor, involving all the residues within a sphere of R = 10 Å centered in the geometrical center of LPS into the flexible docking part of the receptor. The binding energies between the ligands and *h*TRL4/MD2 calculated by the flexible molecular docking AutoDock Vina 1.1.2 are summarized in Figure 4.

As can be seen from Figure 4, the top-scoring binding energies were predicted for compounds **19**, **20**, **22** and **23**, which may be due to their naphtyl or tetrahydronapthyl moieties exhibiting relatively stronger interactions in mostly lipophilic active site of *h*TRL4/MD2. Nonetheless, the best in vitro agonistic activity for *h*TRL4/MD2 receptor was shown by compound **17** containing 4-chlorophenyl moiety, giving the molecule a lower lipophilicity in comparison to **19** or **21**.

**Figure 5:**
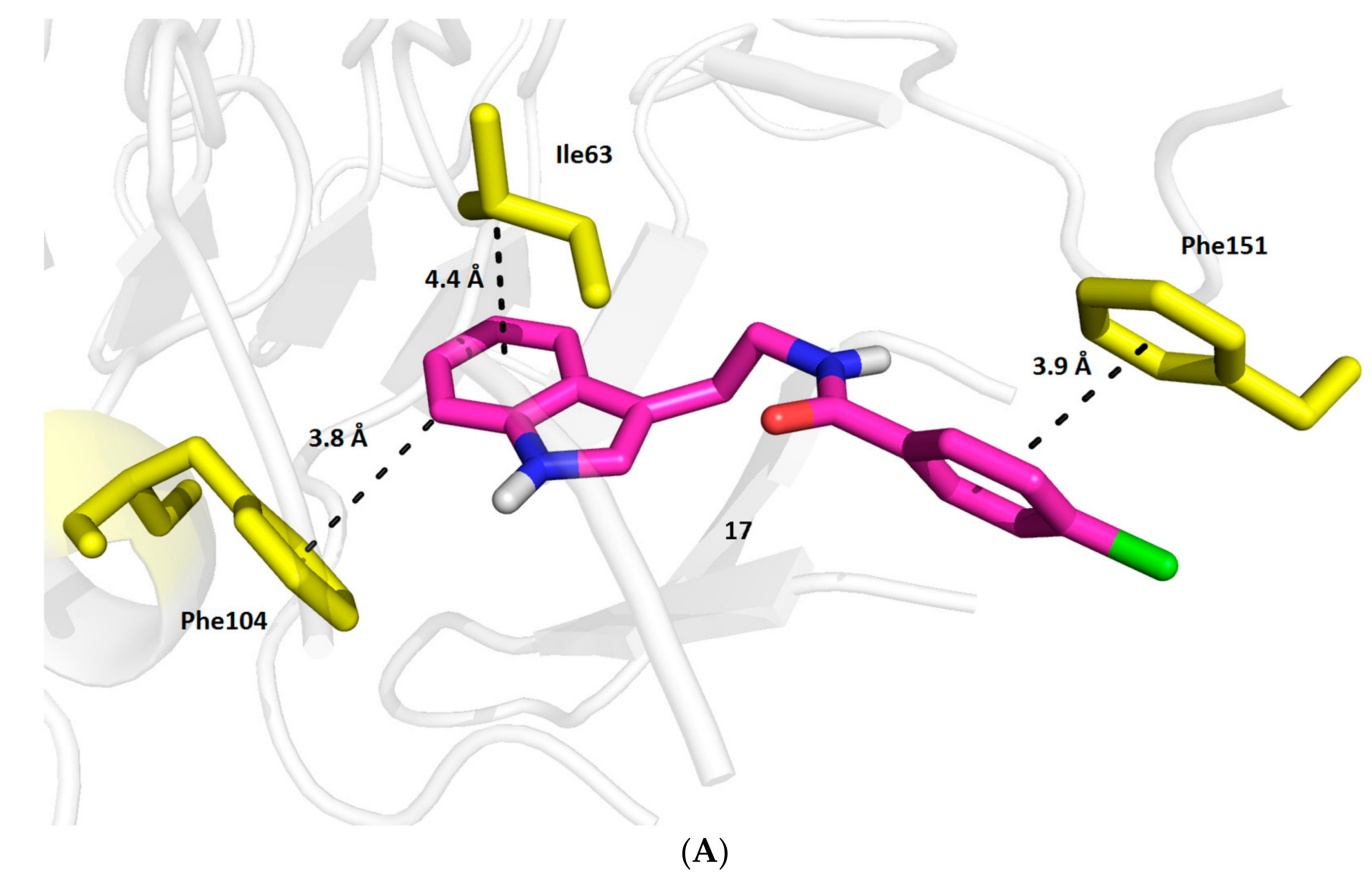

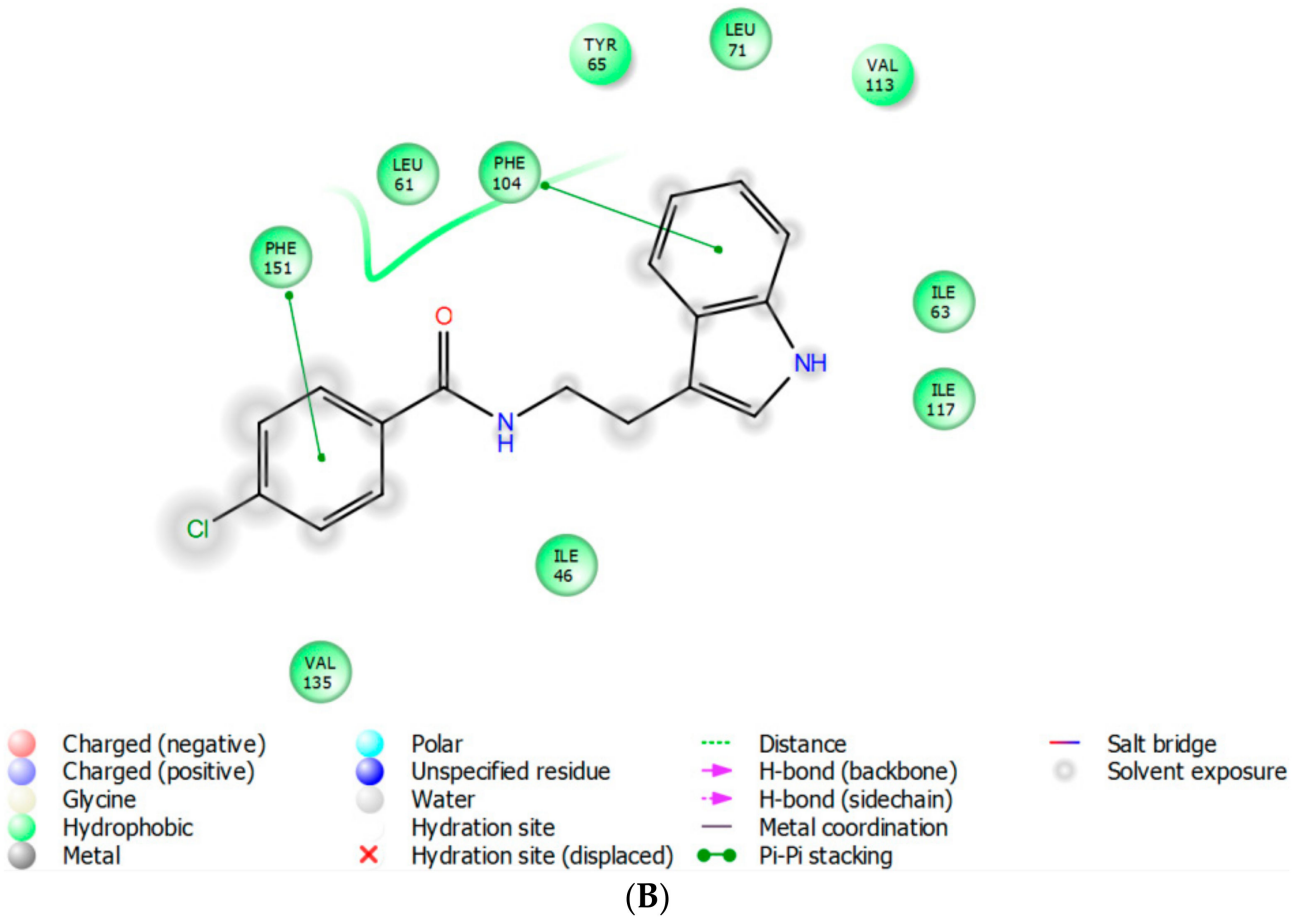
Biding mode of compound **17** in *h*TRL4/MD2 (PDB ID: 4G8A) obtained by flexible molecular docking; (**A**) 3D visualization (The carbon atoms of the ligand are colored in magenta, the aminoacid residues are colored in yellow); (**B**) 2D model.

Molecular modeling revealed that **17** is predominantly stabilized in the *h*TRL4/MD2 active site by weak interactions with aromatic residues (Figure 5). The indolyl moiety of **17** interacts by T-shaped π-π coupling with Phe104 (3.8 Å) and this ligand part is additionally anchored in the position by Ile63 (4.4 Å). The 4-chlorophenyl function in the second molecular part of **17** is involved in a slightly shifted π-π stacking with Phe151 (3.9 Å). From the physico-chemical point of view, these interactions, characterized by the calculated Gibbs energy of −9.5 kcal/mol, seems to be rather weak. However as we discovered in this study, the in silico binding energy of LPS to *h*TRL4/MD2 is even lower, −8.7 kcal/mol, although it elicits very strong agonistic response from the receptor. Another active site residues in *h*TRL4/MD2 are located in a longer distance from **17** and they are not considered as significantly contributing to the complex stabilization.

### 2.4. Molecular Dynamic Study

In order to evaluate the binding modes and binding energy estimates obtained by flexible molecular docking in AutoDock Vina 1.1.2, a molecular dynamic study (MD) was performed in Gromacs 5.1.2 using OPLSAA force field. Accelerated performance in the MD study was achieved by applying a supercomputer equipped with computing nodes with 16 CPUs (Intel Xeon E5-2470) and 1 GPU (NVIDIA Tesla K20m). Using a self-developed bash script we carried out 10 ns simulations of all the 12 ligand-protein complexes in water. The hTRL4/MD2 (PDB ID: 4G8A) model with the docked ligands were paremetrized by Topobuild 1.3 program, hydrated in a dodecahedron solvent box, neutralized by Na^+^/Cl^−^ ions as appropriate and geometrically optimized by the steepest descent algorithm. Then, these ligand-receptor complexes were simulated for 10 ns at 300 K in a parallel calculation mode. The obtained MD trajectories and binary input files (i.e., xtc and tpr files) were adjusted by trjconv function, sampled with 250 ps frequency, and subsequently analyzed by g_mmpbsa software to obtain the binding free energy estimates by MM/PBSA method [27,28]. The obtained energies are summarized in Table 2.

All the resulting MD trajectories were visually checked whether the ligand-receptor complexes are stably bonded during the simulations. In all the complexes, generally stable interactions were observed, which is illustrated by the average RMSDs for the ligands fluctuating between 0.064–0.186 nm. It seems that larger values of RMSD for the ligand conformations are associated with larger deviations of the MM/PBSA binding energies, but it is important to take into consideration that the ligand-receptor complex can move as a whole, which leads to an increased RMSD for ligands, while the complex can stay stable. In Supporting Information, overlaid ligand conformations obtained in the MD simulations are given (frames sampled with frequency of 25 ps (Figures S14–S25)). From the MD simulations, it was also obvious that the MD2 domain, which is not covalently bounded to the TLR4 domain, can move in some cases slightly further from the starting position (e.g., for **21**). Such structural changes may have significant impact on *h*TLR4/MD2 dimerization, and, consequently, on eliciting the agonistic receptor response. The strongest binding energy within the MD study was calculated for **24**, **14** and **17** (a record of the MD simulation for **17** is given in Supporting Information (Video 1)). In comparison with the binding energies predicted by molecular docking, compounds **19** and **20** did not exhibit so strong interactions with hTRL4/MD2 in the MD study. Nonetheless, the MM/PBSA binding energies seem to be closer to the experimental data, although the correct ranking of compounds according to their in vitro activities for *h*TLR4/MD2 cannot be achieved by these MM/PBSA energies.

### 2.5. Cytotoxicity, In Vitro and Ex Vivo Effects

The toxicity was estimated on the cell cultures in order to exclude the most toxic motifs from further development and to test whether the compounds might be used in the functional cell-model of TLR4. Standard MTT assay (Sigma Aldrich, Damrstadt, Germany) was applied according to the manufacturer’s protocol on the CHO-K1 (Chinese hamster ovary, ECACC, Salisbury, UK).

The IC_50_ values were calculated from the control-subtracted triplicates using non-linear regression (four parameters) of GraphPad Prism 5 software (GraphPad Software, La Jolla, CA, USA). Final IC_50_ and SEM value was obtained as a mean of 2–3 independent measurements (see Table 3). The results clearly shows that our compounds’ cytotoxic effect is relatively low (IC_50_ ranging from 98–892 μM, thus they can be used in subsequent experiments using *h*TL4R4/MD2 cell model (Section 2.6).

### 2.6. In Vitro Activity for hTLR4/MD2

**Figure 6:**
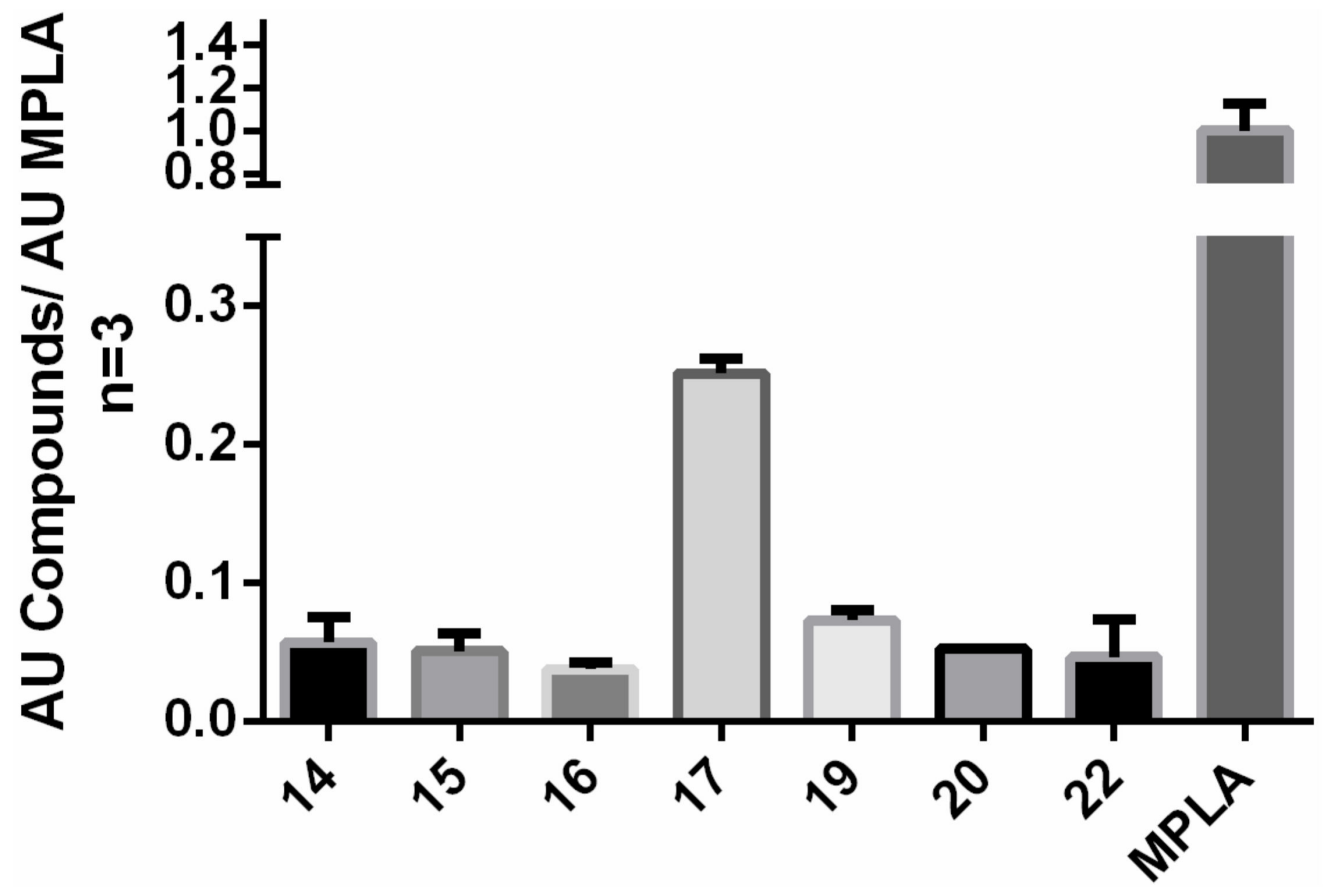
*h*TLR4/MD2 in vitro evaluation. Values normalized to the activity of MPLA expressed as the Mean ± standard error of mean (*n* = 3). For conditions see the experimental section.

We have tested each of the newly-synthesized compound at 10 µM (10% of lowest cytotoxic IC_50_, see Section 2.5) for an internal activity on a cell line stably expressing the *h*TLR4/MD2 receptor and found that compound **17** reaches more than 20% and Compound **22** approximately showed 10% activity of MPLA (Figure 6). Compounds **24** and **25** weren’t evaluated due to stability issues of the compounds (unwanted degradations from crystals to amorphous state). Thus we have provide a proof of concept of the *h*TLR4/MD2 agonists’ rational design and by that we have revealed, until now unknown class of ligands, which has promising intrinsic activity and are easily synthesizable.

### 2.7. Surface Plasmon Resonance (SPR)

**Figure 7:**
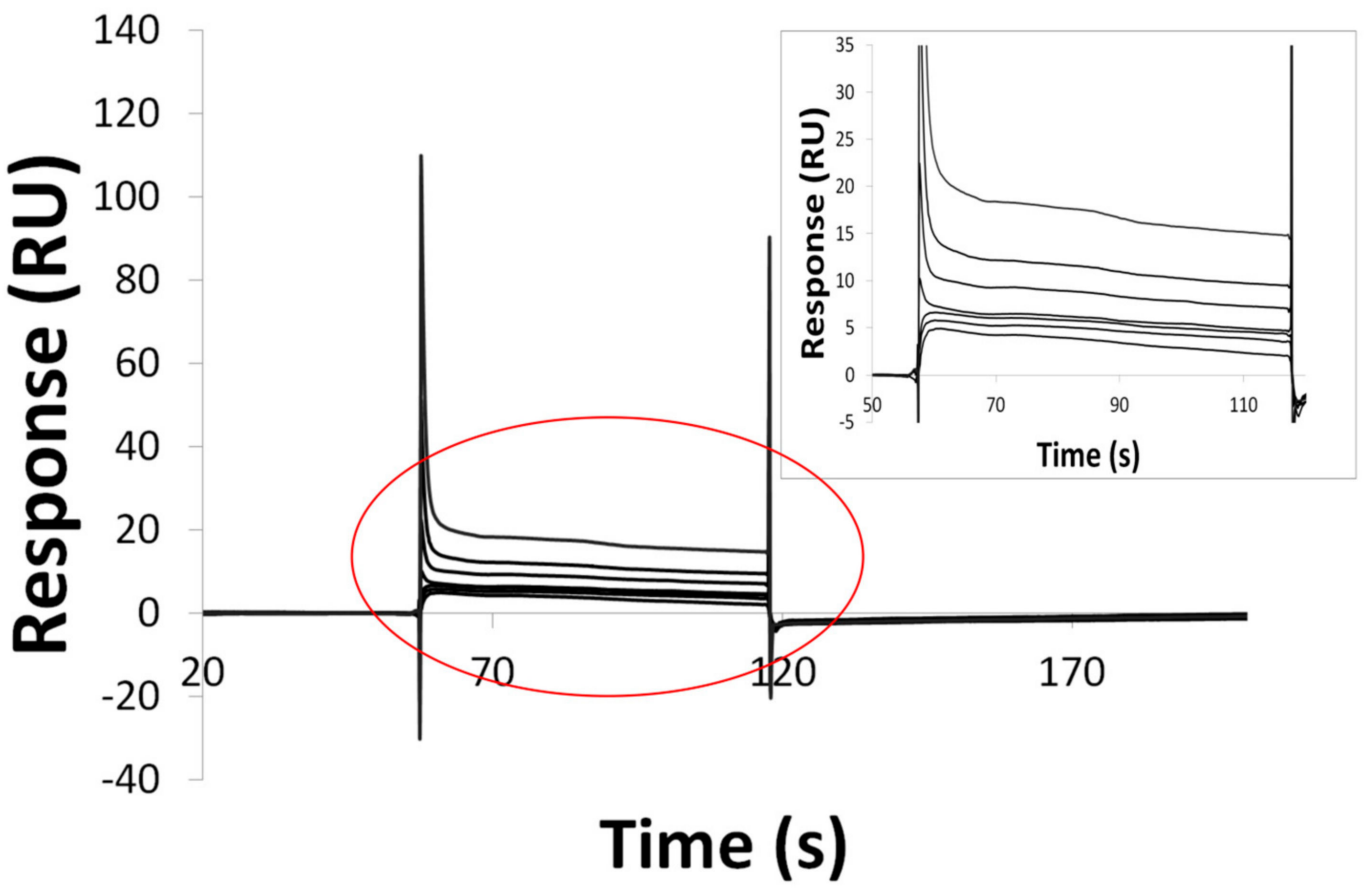
SPR experiment—Binding of compound **17** to the chip with immobilized *h*TRL4/MD2 complex. Compound **17** was injected at concentrations from 6.25 to 200 µM (from bottom to the top –inset represents same data on magnified scale for better discernibility).

To shed light on the prediction capabilities of vHTPS, direct binding of the best compound from the in vitro study (**17**) to the *h*TRL4/MD2 complex was investigated through Biospecific Interaction Analysis using the SPR-based biosensor Biacore T200 (GE Healthcare, Little Chalfont, UK). Recombinant *h*TRL4/MD2 complex (R&D Systems, Abingdon, UK) was immobilized on the chip, and the direct binding to the *h*TRL4/MD2 complex was confirmed (Figure 7).

We think that it is reasonable to suggest, because of the similarity of presented compounds, that all other compounds are interacting with the *h*TRL4/MD2 complex in a similar way. Compound 17 was further analyzed using a series of concentrations from 6.25 up to 200 μM (binary dilution). The clear dose-response binding of compound **17** was observed (Figure 7), with rapid dissociation rates. K_D_ was determined at 7.47 × 10^−7^ M.

### 2.8. Ex Vivo Stimulation of IL-6 Production

Important factor in adjuvants development is wheter the compounds are capable to elicit immune response not only on reporter cells but on real immunocompetent cels. We have chosen ex vivo study on human Periferal blood mononuclear cells (PBMCs) to evaluate the ability to produce an interleukin 6 (IL-6). IL-6 plays critical role in the innate imunity possessing both pro and anti-inflamatory properties. We have collected the blood samples from healthy donors. From those blood samples we had harvested PBMCs by standard protocol using Histopaque 1077 density gradient. PBMC specimens were frozen and kept in liquid nitrogen. The cells were incubated for 24 h with 10 µM (10% of lowest cytotoxic IC_50_—see the Section 2.5 and Section 2.6) of each compound and the ability to elicit IL-6 production was assessed. Synthetic MPLA (MPLAs) in equimolar concentration (10 μM) was used as a positive control and LPS from *Rhodobacter sphaeroides* 100 ng/mL (LPS-RS) as a negative control. IL-6 production was quantified by ELISA commercial kit (Sigma Aldrich) and results were interpreted by GraphPad Prism using Nonlinear Fit of RIA data function. We observed, that the Compounds **14** and **22** were able to significantly induce production of IL-6 which is in line with the in-silico prediction. On the other hand hit compound from the in vitro study (**17**) did not shown any effect on the IL-6 production. Effect on other pro-inflamatory cytokines (e.g., IL-1) was not determined in this study and will be subject of ongoing study in order to explain discrepancy among in silico, in vitro and ex vivo findings. Furthermore study evaluating the influence of compounds on Th1/Th2/Th17 bias will be needed to thoroughly assess their adjuvant and immunomodulating potential. Nevertheless, despite such necesarry and laborious tasks novel TLR4 agonists and potential vaccine adjuvants have been found.

The results are sumarized in Table 4. All the experiment were approved by the Ethical Committee and informed consent was obtained from all subjects (healthy volounteers).

## 3. Experimental Section

### 3.1. Virtual Screening

In this study a combined ligand and structure based virtual screening with utilization of a peta-flops-scale supercomputer (www.it4i.cz) has been performed to reveal promising hit structures able to modulate *h*TLR4/MD2 receptor (PDB ID: 4G8A). In the first stage, a random selection of 130,000 compounds having MW ≤ 500, xlogp ≤ 7, HBD ≤ 5, and HBA ≤ 10 was downloaded from the online ZINC database and processed in Screening Assistant 2 program. From this custom virtual ligand library, 9700 structures exhibiting Tanimoto similarity index higher than 0.7 towards carbamazepine, fentanyl, morphine, oxycodone, morphine-3-glucuronide, levorphanol, buprenorphine, oxcarbazepine, ethanol, pethidine, methadone, mianserin, imipramine, eritoran, cyclobenzaprine, naltrexone, ibudilast, ketotifen, propentofylline, amitriptyline, naloxone were selected. Additional 300 ligands were obtained from our in house compound database or designed according to the literature [10,12,13,14,15,16,17]. In total, 10,000 ligands were submitted to structure-based virtual screening by flexible molecular docking.

In structure based virtual screening, a 3D X-ray model of *h*TLR4/MD2 (PDB ID: 4G8A), resolution: 2.4 Å) was downloaded from the online PDB database (http://www.rcsb.org/) and prepared for molecular docking by MGL Tools utilities. The model *h*TLR4/MD2 (PDB ID: 4G8A) was engineered by Ohto et al. to correct three mutations (ASP299 to GLY299, THR399 to ILE399, and ARG56 to GLY56) [29]. The preparation of the receptor involved removal of the surplus copies of the enzyme chains, water molecules, non-bonded inhibitors and cofactors, addition of polar hydrogens and merging of non-polar ones. Default Gasteiger charges were assigned to all the atoms. The flexible part of the *h*TLR4/MD2 receptor was determined by a spherical selection of residues (R = 11 Å) approximately around the geometrical center of the co-crystalized molecule of LPS (*x* = −14.925, *y* = −19.741, *z* = −10.93), which corresponds to the center of the receptor binding site. In the same point the center of the grid box of 33 × 33 × 33 Å was positioned. The rotatable bonds in the flexible residues were detected automatically by AutoDock Tools 1.5.4 program. The flexible receptor part contained 32 residues (VAL24, CYS25, ILE32, SER33, TYR34, ILE46, VAL48, CYS51, ILE52, GLU53, LEU54, SER57, LEU61, ILE63, PHE76, LEU78, ILE80, GLU92, PHE119, SER120, PHE121, PHE126, TYR131, LYS132, CYS133, VAL134, VAL135, LEU149, GLU150, PHE151, VAL152, ILE153).

The complete set of 10,000 ligands was protonized as appropriate for pH = 7.4 in OpenBabel 2.3.3 and converted into pdbqt format in AutoDock Tools 1.5.4 program in the same way as the flexible hTLR4/MD2 enzyme part. By means of a bash-based script for efficient parallelization, all the ligands were docked in hTLR4/MD2 using AutoDock Vina 1.1.2 program as a molecular docking engine. Basically, AutoDock Vina combines a Markov chain Monte Carlo like method for the global search and a Broyden-Fletcher-Goldfarb-Shano gradient approach for the local search [30]. It is a type of memetic algorithm based on interleaving stochastic and deterministic calculations [26,31]. Each molecular docking task was performed with the exhaustiveness parameter set to 16, employing 16 CPU in parallel multithreading. The top scoring candidates were chosen according to the minimum predicted Gibbs binding energy. The graphic representations of the docked poses were rendered in PyMOL 1.6. (The PyMOL Molecular Graphics System, Version 1.6. Schrödinger, LLC).

### 3.2. Molecular Docking Study

The receptor *h*TLR4/MD2 (PDB ID: 4G8A*)* for flexible molecular docking in AutoDock Vina was prepared in the same way as mentioned in Section 3.1 on the virtual screening. The models of the newly prepared compounds **14**–**25** were firstly drawn in HyperChem 8.0 (HyperCube Inc., Gainesville, FL, USA), then manually protonated as suggested by MarvinSketch 6.2.0. software, geometrically optimized by semi-empirical quantum-chemistry PM3 method and stored as pdb files. The structures of these ligands were processed for docking in a similar way as the flexible parts of the *h*TLR4/MD2 receptor by AutoDock Tools 1.5.4 program.The same setting molecular docking (i.e., gridbox parameters) as in the virtual screening was applied. Each docking task was repeated 10 times with the exhaustiveness parameter set to 16, employing 16 CPU in parallel multithreading. From the obtained results, the solutions reaching the minimum predicted Gibbs binding energy were taken as the top-scoring modes. The graphic representations of the docked poses were rendered in PyMOL 1.6 (Schroedinger, Manheim, Germany), 2D diagrams were generated using Maestro 10.1 (Schroedinger, Manheim, Germany).

### 3.3. Molecular Dynamics

MM/PBSA binding energies of the ligand-receptor complexes (**14**–**25**), obtained in the molecular docking study, were predicted employing molecular dynamic simulations in Gromacs 5.1.2 and g_mmpbsa software. All the chemical structures were parameterized by means of OPLSAA force field in Topobuild 1.3. program). Each molecular system contained the *h*TLR4/MD2 (PDB ID: 4G8A) with the docked compounds, water molecules (SPC/E, single point charge), and Na^+^/Cl^−^ ions to neutralize the total charge in a dodecahedron body shape. Then the systems were minimized by the steepest descent algorithm to release any close contacts, equilibrated for 100 ps under canonical NVT conditions (N—number of particles, V—the system volume, T—temperature) at 300 K and simulated for 10 ns under NPT (P—pressure) conditions at 300 K. In the production MD run, the step of 2 fs, time constant of 0.1 ps with modified Berendsen thermostat, pressure coupling by a semi-isotropic Parrinello-Rahman barostat with the time constant of 2 ps and 1 bar reference pressure and holonomic constraints (i.e., LINCS algorithm) were applied. The neighbor list for the calculation of nonbonded interactions was updated every five time steps with the cutoff of 1.2 nm. A twin range cutoff of 1.4 nm was used for both Coulomb and Lennard-Jones interactions. Particle Mesh Ewald (PME) method was used for the treatment of long-range electrostatic interactions.

The obtained MD trajectories were adjusted by trjconv and sampled to skip every 25 frames. These altered trajectory files were processed in g_mmpsa to calculate polar, apolar, and vacuum potential energy contributions to free binding energy estimates between the ligands and *h*TLR4/MD2. The resulting data were next processed by MmPbSaStat.py script developed by Rashmi Kumari and Andrew Lynn (http://rashmikumari.github.io/g_mmpbsa/) to obtain the binding energy estimates and their standard sample deviations. Using rmsdist subroutine in Gromacs, root mean square distances (RMSD) for the ligand movements in the complexes during the MD simulations were calculated. All the molecular dynamic and MM/PBSA calculations were carried out in supercomputers (www.it4i.cz) equipped with nodes containing 16 CPUs (Intel Xeon E5-2470, Intel) and 1 GPU (NVIDIA Tesla K20m).

### 3.4. Chemistry General Methods

All commercial reagents and dry solvents used were of the highest available purity from Sigma-Aldrich (Darmstadt, Germany). All reactions were performed under an atmosphere of nitrogen, unless otherwise noted. For flash column chromatography on silica gel, Kieselgel 60 (0.063–0.200 mm, 70–230 mesh, Fluka) was used. The solvents for column chromatography were purchased from Penta chemicals Co. (Prague, Czech Republic), and solvents for LC/MS analyses were obtained in LC-MS grade purity from Sigma-Aldrich (Darmstadt, Germany). Thin layer chromatography was run on silica gel 60 F_254_ analytical plates (Merck, Darmstadt, Germany); mobile phase: heptane/EtOAc 1/1; heptane/EtOAc 2/1); detection was carried out with ultraviolet light (254 nm). Melting points were recorded on a Stuart SMP30 Melting Point Apparatus (Cole-Parmer, Stone, UK) and are uncorrected. The ^1^H NMR and ^13^C NMR spectra were recorded in DMSO-*d*_6_ solution at ambient temperature on a S500 spectrometer (Varian, Palo Alto, CA, USA); 499.87 MHz for ^1^H and 125.71 MHz for ^13^C). Chemical shifts, *δ*, are given in parts per million (ppm), and spin multiplicities are given as s (singlet), d (doublet), t (triplet), or m (multiplet). Coupling constants, *J*, are expressed in hertz (Hz). For ^1^H *δ* relative to DMSO-*d*_6_ (*δ* = 2.50) and for ^13^C relative to DMSO-*d*_6_ (*δ* = 39.43). HRMS spectra were determined by Q Exactive Plus hybrid quadrupole-orbitrap spectrometer (ThermoFisher, Bremen, Germany).

#### 3.4.1. General Procedure for Synthesis of Compounds **14**–**21** by Reaction of Commercially Available Acyl Chlorides with Tryptamine (Procedure A)

To the solution of tryptamine (**1**, 1.87 mM) in dry CH_2_Cl_2_ (8 mL), that had been pre-cooled to 0 °C, the Et_3_N (3.74 mM) and acyl chloride (**2**–**9**, 2.43 mM) were gradually added. The resulting mixture was stirred for 10 min at 0 °C and then at room temperature for a further 50 min. The course of reactions was monitored by TLC. Subsequently the solvent was evaporated under vacuum and the residue was chromatographed on silica gel, eluent heptane–EtOAc (1:1) (for compounds **14**–**16**) and heptane–EtOAc (2:1) (for compounds **17**–**21**).

*N-(2-(1H-Indol-3-yl)ethyl)benzamide* (**14**)*.* Compound **1** was treated with benzoyl chloride (**2**) according to the above procedure A to give the desired product **14** in the form of white crystals (81%); m.p.: 141.2–142.8 °C. ^1^H-NMR (DMSO-*d*_6_) *δ* 2.95–2.98 (m, 2H, CH_2_), 3.54–3.58 (m, 2H, CH_2_), 6.97–7.00 (m, 1H, ArH), 7.06–7.09 (m, 1H, ArH), 7.18–7.19 (m, 1H, ArH), 7.35 (d, *J* = 8.1 Hz, 1H, ArH), 7.44–7.47 (m, 2H, 2 × ArH), 7.50–7.53 (m, 1H, ArH), 7.59 (d, *J* = 7.8 Hz, 1H, ArH), 7.85–7.87 (m, 2H, 2 × ArH), 8.61 (t, *J* = 5.5 Hz, 1H, NH), 10.81 (s, 1H, NH); ^13^C-NMR (DMSO-*d*_6_) *δ* 25.10, 40.12, 111.27, 111.81, 118.12, 118.19, 120.81, 122.50, 127.02, 127.19, 128.13, 130.90, 134.62, 136.15, 166.01 ppm. HRMS (HESI^+^): [M + H]^+^: calculated for C_17_H_17_N_2_O^+^ (*m*/*z*): 265.1335; found: 265.1331.

*N-(2-(1H-Indol-3-yl)ethyl)-4-methylbenzamide* (**15**). Compound **1** was treated with 4-methylbenzoyl chloride (**3**) according to the above procedure A to give the desired product **15** in the form of a white crystals (92%); m.p.: 126.8–128.2 °C. ^1^H-NMR (DMSO-*d*_6_) *δ* 2.35 (s, 3H, CH_3_), 2.94–2.97 (m, 2H, CH_2_), 3.52–3.57 (m, 2H, CH_2_), 6.97–7.00 (m, 1H, ArH), 7.06–7.09 (m, 1H, ArH), 7.18–7.19 (m, 1H, ArH), 7.26 (d, *J* = 8.1 Hz, 2H, 2 × ArH), 7.35 (d, *J* = 8.1 Hz, 1H, ArH), 7.59 (d, *J* = 7.8 Hz, 1H, ArH), 7.77 (d, *J* = 8.2 Hz, 2H, 2 × ArH), 8.53 (t, *J* = 5.6 Hz, 1H, NH), 10.81 (s, 1H, NH); ^13^C-NMR (DMSO-*d*_6_) *δ* 20.83, 25.15, 40.08, 111.26, 111.85, 118.11, 118.20, 120.81, 122.47, 127.04, 127.20, 128.65, 131.83, 136.15, 140.71, 165.89 ppm. HRMS (HESI^+^): [M + H]^+^: calculated for C_18_H_19_N_2_O^+^ (*m*/*z*): 279.1492; found: 279.1488.

*N-(2-(1H-Indol-3-yl)ethyl)-4-methoxybenzamide* (**16**). Compound **1** was treated with 4-methoxybenzoyl chloride (**4**) according to the above procedure A to give the desired product **16** in the form of a white crystals (87%); m.p.: 132.8–134.3 °C. ^1^H-NMR (DMSO-*d*_6_) *δ* 2.93–2.96 (m, 2H, CH_2_), 3.51–3.55 (m, 2H, CH_2_), 3.81 (s, 3H, OCH_3_), 6.97–7.01 (m, 3H, 3 × ArH), 7.05–7.09 (m, 1H, ArH), 7.17–7.18 (m, 1H, ArH), 7.34 (d, *J* = 8.1 Hz, 1H, ArH), 7.59 (d, *J* = 7.8 Hz, 1H, ArH), 7.84 (d, *J* = 8.8 Hz, 2H, 2 × ArH), 8.46 (t, *J* = 5.5 Hz, 1H, NH), 10.81 (s, 1H, NH); ^13^C-NMR (DMSO-*d*_6_) *δ* 25.21, 40.06, 55.21, 111.26, 111.88, 113.32, 118.10, 118.21, 120.80, 122.46, 126.85, 127.19, 128.82, 136.14, 161.31, 165.49 ppm. HRMS (HESI^+^): [M + H]^+^: calculated for C_18_H_19_N_2_O_2_^+^ (*m*/*z*): 295.1441; found: 295.1436.

*N-(2-(1H-Indol-3-yl)ethyl)-4-chlorobenzamide* (**17**). Compound **1** was treated with 4-chlorobenzoyl chloride (**5**) according to the above procedure A to give the desired product **17** in the form of a white crystals (66%); m.p.: 150.6–152.0 °C. ^1^H-NMR (DMSO-*d*_6_) *δ* 2.95 (t, *J* = 7.5 Hz, 2H, CH_2_), 3.52–3.57 (m, 2H, CH_2_), 6.96–7.00 (m, 1H, ArH), 7.05–7.08 (m, 1H, ArH), 7.17–7.19 (m, 1H, ArH), 7.34 (d, *J* = 8.1 Hz, 1H, ArH), 7.54 (d, *J* = 8.6 Hz, 2H, 2 × ArH), 7.58 (d, *J* = 7.8 Hz, 1H, ArH), 7.87 (d, *J* = 8.6 Hz, 2H, 2 × ArH), 8.69 (t, *J* = 5.6 Hz, 1H, NH), 10.81 (s, 1H, NH); ^13^C-NMR (DMSO-*d*_6_) *δ* 25.01, 40.19, 111.27, 111.73, 118.12, 118.16, 120.81, 122.52, 127.16, 128.22, 128.97, 133.32, 135.74, 136.14, 164.94 ppm. HRMS (HESI^+^): [M + H]^+^: calculated for C_17_H_16_ClN_2_O^+^ (*m*/*z*): 299.0946; found: 299.0941.

*N-(2-(1H-Indol-3-yl)ethyl)-3,4-dichlorobenzamide* (**18**). Compound **1** was treated with 3,4-dichlorobenzoyl chloride (**6**) according to the above procedure A to give the desired product **18** in the form of a white crystals (75%); m.p.: 112.4–113.9 °C. ^1^H-NMR (DMSO-*d*_6_) *δ* 2.96 (t, *J* = 7.4 Hz, 2H, CH_2_), 3.53–3.57 (m, 2H, CH_2_), 6.96–7.00 (m, 1H, ArH), 7.05–7.08 (m, 1H, ArH), 7.18–7.19 (m, 1H, ArH), 7.34 (d, *J* = 8.1 Hz, 1H, ArH), 7.57 (d, *J* = 7.9 Hz, 1H, ArH), 7.75 (d, *J* = 8.4 Hz, 1H, ArH), 7.83 (dd, *J* = 2.1, 8.4 Hz, 1H, ArH), 8.07 (d, *J* = 2.0 Hz, 1H, ArH), 8.80 (t, *J* = 5.5 Hz, 1H, NH), 10.82 (s, 1H, NH); ^13^C-NMR (DMSO-*d*_6_) *δ* 24.88, 40.32, 111.29, 111.64, 118.13, 120.82, 122.57, 127.15, 127.38, 129.03, 130.56, 131.10, 133.74, 134.89, 136.14, 163.72 ppm. HRMS (HESI^+^): [M + H]^+^: calculated for C_17_H_15_Cl_2_N_2_O^+^ (*m*/*z*): 333.0556; found: 333.0553.

*N-(2-(1H-Indol-3-yl)ethyl)-2-naphthamide* (**19**). Compound **1** was treated with 2-naphthoyl chloride (**7**) according to the above procedure A to give the desired product **19** in the form of a white crystals (92%); m.p.: 193.2–195.0 °C. ^1^H-NMR (DMSO-*d*_6_) *δ* 3.02 (t, *J* = 7.5 Hz, 2H, CH_2_), 3.61–3.65 (m, 2H, CH_2_), 6.98–7.02 (m, 1H, ArH), 7.07–7.10 (m, 1H, ArH), 7.22–7.24 (m, 1H, ArH), 7.36 (d, J = 8.1 Hz, 1H, ArH), 7.57–7.64 (m, 3H, 3 × ArH), 7.95–8.03 (m, 4H, 4 × ArH), 8.47 (s, 1H, ArH), 8.80 (t, J = 5.6 Hz, 1H, NH), 10.84 (s, 1H, NH); ^13^C-NMR (DMSO-*d*_6_) δ 25.15, 40.28, 111.30, 111.85, 118.15, 118.24, 120.84, 122.55, 124.10, 126.59, 127.25, 127.40, 127.52, 127.73, 128.70, 132.00, 132.08, 133.97, 136.17, 166.09 ppm. HRMS (HESI^+^): [M + H]^+^: calculated for C_21_H_19_N_2_O^+^ (*m*/*z*): 315.1492; found: 315.1487.

*N-(2-(1H-Indol-3-yl)ethyl)cyclohexanecarboxamide* (**20**). Compound **1** was treated with cyclohexanecarbonyl chloride (**8**) according to the above procedure A to give the desired product **20** in the form of a white crystals (59%); m.p.: 104.8–106.3 °C. ^1^H-NMR (DMSO-*d*_6_) *δ* 1.12–1.24 (m, 4H, 2 × CH_2_), 1.29–1.37 (m, 2H, CH_2_), 1.65–1.71 (m, 4H, 2 × CH_2_), 2.04–2.10 (m, 1H, CH), 2.80 (t, *J* = 7.4 Hz, 2H, CH_2_), 3.28–3.32 (m, 2H, CH_2_), 6.95–6.99 (m, 1H, ArH), 7.04–7.07 (m, 1H, ArH), 7.11–7.13 (m, 1H, ArH), 7.33 (d, *J* = 8.1 Hz, 1H, ArH), 7.53 (d, *J* = 7.8 Hz, 1H, ArH), 7.76 (t, *J* = 5.5 Hz, 1H, NH), 10.78 (s, 1H, NH); ^13^C-NMR (DMSO-*d*_6_) *δ* 25.14, 25.22, 25.41, 29.16, 39.19, 43.96, 111.21, 111.80, 118.03, 118.15, 120.75, 122.44, 127.15, 136.11, 174.90 ppm. HRMS (HESI^+^): [M + H]^+^: calculated for C_17_H_23_N_2_O^+^ (*m*/*z*): 271.1805; found: 271.1799.

*N-(2-(1H-Indol-3-yl)ethyl)-1-naphthamide* (**21**). Compound **1** was treated with 1-naphthoyl chloride (**9**) according to the above procedure A to give the desired product **21** in the form of a white crystals (80%); m.p.: 164.5–166.5 °C. ^1^H-NMR (DMSO-*d*_6_) *δ* 3.04 (t, *J* = 7.4 Hz, 2H, CH_2_), 3.63–3.67 (m, 2H, CH_2_), 7.00–7.03 (m, 1H, ArH), 7.08–7.11 (m, 1H, ArH), 7.24–7.26 (m, 1H, ArH), 7.38 (d, *J* = 8.1 Hz, 1H, ArH), 7.51–7.57 (m, 4H, 4 × ArH), 7.64 (d, *J* = 7.8 Hz, 1H, ArH), 7.95–8.00 (m, 2H, 2 × ArH), 8.14–8.16 (m, 1H, ArH), 8.64 (t, *J* = 5.6 Hz, 1H, NH), 10.87 (s, 1H, NH); ^13^C-NMR (DMSO-*d*_6_) *δ* 25.06, 39.93, 111.30, 111.74, 118.15, 118.26, 120.84, 122.70, 124.85, 124.87, 125.41, 126.06, 126.48, 127.24, 128.03, 129.48, 129.66, 133.01, 135.09, 136.20, 168.39 ppm. HRMS (HESI^+^): [M + H]^+^: calculated for C_21_H_19_N_2_O^+^ (*m*/*z*): 315.1492; found: 315.1486.

#### 3.4.2. General Procedure for Synthesis of Compounds **22**–**25** by Reaction of Commercially Available Carboxylic Acids with Tryptamine (Procedure B)

To the solution of carboxylic acid **10**–**13** (2.43 mM) in dry CH_2_Cl_2_ (5.5 mL) that had been pre-cooled to 0 °C, were gradually added the catalytic amount of DMF (1–2 drop) and oxalyl chloride (7.29 mM). The resulting mixture was stirred for 5 min at 0 °C and then at room temperature for a further 55 min. Subsequently the solvent was evaporated under vacuum and the crude product was used further.

To solution of tryptamine (**1**, 1.87 mM) in dry CH_2_Cl_2_ (5 mL) were gradually added at 0 °C the Et_3_N (3.74 mM) and solution of crude product in dry CH_2_Cl_2_ (3 mL). The resulting mixture was stirred for 10 min at 0 °C and then at room temperature for a further 50 min. Subsequently the solvent was evaporated under vacuum and the residue was chromatographed on silica gel, using heptane–EtOAc (2:1) as eluent.

*N-(2-(1H-Indol-3-yl)ethyl)-1,2,3,4-tetrahydronaphthalene-2-carboxamide* (**22**). Product **22** was prepared from 1,2,3,4-tetrahydro-2-naphthoic acid (**10**) according to the above procedure B in the form of a white crystals (81%); m.p.: 123.2–125.2 °C. ^1^H-NMR (DMSO-*d*_6_) *δ* 1.64–1.73 (m, 1H, CH), 1.91–1.95 (m, 1H, CH), 2.46–2.49 (m, 1H, CH), 2.69–2.87 (m, 6H, 3 x CH_2_), 3.35–3.40 (m, 2H, CH_2_), 6.97–7.00 (m, 1H, ArH), 7.04–7.09 (m, 5H, 5 × ArH), 7.15–7.17 (m, 1H, ArH), 7.35 (d, *J* = 8.1 Hz, 1H, ArH), 7.56 (d, *J* = 7.9 Hz, 1H, ArH), 7.99 (t, *J* = 5.6 Hz, 1H, NH), 10.81 (s, 1H, NH); ^13^C-NMR (DMSO-*d*_6_) *δ* 25.16, 26.21, 28.13, 31.77, 39.39, 40.33, 111.24, 111.77, 118.07, 118.18, 120.78, 122.53, 125.41, 125.43, 127.18, 128.46, 128.71, 135.46, 135.54, 136.13, 174.35 ppm. HRMS (HESI^+^): [M + H]^+^: calculated for C_21_H_23_N_2_O^+^ (*m*/*z*): 319.1805; found: 319.1800.

*N-(2-(1H-Indol-3-yl)ethyl)-5,6,7,8-tetrahydronaphthalene-2-carboxamide* (**23**). Product **23** was prepared from 5,6,7,8-tetrahydro-2-naphthoic acid (**11**) according to the above procedure B in the form of a yellow-white crystals (91%); m.p.: 137.0–139.0 °C. ^1^H-NMR (DMSO-*d*_6_) *δ* 1.72–1.76 (m, 4H, 2 × CH_2_), 2.71–2.78 (m, 4H, 2 × CH_2_), 2.93–2.96 (m, 2H, CH_2_), 3.51–3.55 (m, 2H, CH_2_), 6.97–7.00 (m, 1H, ArH), 7.05–7.08 (m, 1H, ArH), 7.11 (d, *J* = 8.6 Hz, 1H, ArH), 7.16–7.18 (m, 1H, ArH), 7.34 (d, *J* = 8.1 Hz, 1H, ArH), 7.55–7.59 (m, 3H, 3 × ArH), 8.46 (t, *J* = 5.6 Hz, 1H, NH), 10.80 (s, 1H, NH); ^13^C-NMR (DMSO-*d*_6_) *δ* 22.42, 22.51, 25.14, 28.63, 28.70, 40.03, 111.25, 111.86, 118.09, 118.20, 120.79, 122.45, 124.11, 127.20, 127.68, 128.62, 131.80, 136.13, 136.31, 139.79, 166.03 ppm. HRMS (HESI^+^): [M + H]^+^: calculated for C_21_H_23_N_2_O^+^ (*m*/*z*): 319.1805; found: 319.1802.

*N-(2-(1H-Indol-3-yl)ethyl)cycloheptanecarboxamide* (**24**). Product **24** was prepared from cycloheptanecarboxylic acid (**12**) according to the above procedure B in the form of a yellow-white crystals (90%); m.p.: 88.4–90.4 °C. ^1^H-NMR (DMSO-*d*_6_) *δ* 1.34–1.57 (m, 8H, 4 × CH_2_), 1.63–1.73 (m, 4H, 2 × CH_2_), 2.21–2.26 (m, 1H, CH), 2.79 (t, *J* = 7.4 Hz, 2H, CH_2_), 3.28–3.32 (m, 2H, CH_2_), 6.95–6.99 (m, 1H, ArH), 7.04–7.07 (m, 1H, ArH), 7.11–7.13 (m, 1H, ArH), 7.33 (d, *J* = 8.1 Hz, 1H, ArH), 7.53 (d, *J* = 7.8 Hz, 1H, ArH), 7.75 (t, *J* = 5.6 Hz, 1H, NH), 10.78 (s, 1H, NH); ^13^C-NMR (DMSO-*d*_6_) *δ* 25.15, 26.05, 27.83, 31.09, 39.18, 45.55, 111.21, 111.81, 118.03, 118.16, 120.75, 122.42, 127.15, 136.11, 176.01 ppm. HRMS (HESI^+^): [M + H]^+^: calculated for C_18_H_25_N_2_O^+^ (*m*/*z*): 285.1961; found: 285.1958.

*N-(2-(1H-Indol-3-yl)ethyl)-4-methylpentanamide* (**25**). Product **25** was prepared from 4-methylpentanoic acid (**13**) according to the above procedure B in the form of a brown crystals (87%); m.p.: 45.1–47.1 °C. ^1^H-NMR (DMSO-*d*_6_) *δ* 0.85 (d, *J* = 6.6 Hz, 6H, 2 × CH_3_), 1.37–1.41 (m, 2H, CH_2_), 1.44–1.52 (m, 1H, CH), 2.05–2.08 (m, 2H, CH_2_), 2.80 (t, *J* = 7.4 Hz, 2H, CH_2_), 3.30–3.34 (m, 2H, CH_2_), 6.96–6.99 (m, 1H, ArH), 7.04–7.08 (m, 1H, ArH), 7.12–7.14 (m, 1H, ArH), 7.33 (d, *J* = 8.1 Hz, 1H, ArH), 7.53 (d, *J* = 7.8 Hz, 1H, ArH), 7.88 (t, *J* = 5.5 Hz, 1H, NH), 10.80 (s, 1H, NH); ^13^C-NMR (DMSO-*d*_6_) *δ* 22.20, 25.16, 27.11, 33.44, 34.25, 39.32, 111.22, 111.79, 118.06, 118.14, 120.76, 122.45, 127.14, 136.12, 172.02 ppm. HRMS (HESI^+^): [M + H]^+^: calculated for C_16_H_23_N_2_O^+^ (*m*/*z*): 259.1805; found: 259.1803.

### 3.5. In Vitro hTLR4/MD2 Activity Assessment

Aliquots of 20 µL of each compound was added into the well containing 25000 HEK-Blue™-*h*TLR4 cells (Invivogen, Toulouse, France) and the final concentration of compound in each well was 10 µM. Each concentration was evaluated in triplicate. Synthetic MPLAs (Invivogen) of the same concentration was used as positive control, apyrogenous saline F1 (BBraun, Prague, Czech Republic) was used as negative control. Plates were incubated overnight for 16 h at 37 °C 5% CO_2_. After the incubation 20 μL aliquots of supernatant were collected and mixed with 200 μL of SEAP detection media (QuantiBlue™, Invivogen) and incubated for 2 h at 37 °C 5% CO_2_. Absorbance of the solution was measured at 630 nm and activity of each compound expressed as a percentage of an activity of the MPLA of respective concentration was determined (*n* = 3).

### 3.6. Surface Plasmon Resonance Spectroscopy

Surface plasmon resonance (SPR) experiments methodology was adapted from experiments by Svajger et al. [32]. The experiments were performed using a Biacore T200 (GE Healthcare, Little Chalfont, UK) instrument equipped with a Series S—CM5 sensor chip (GE Healthcare). Dulbecco’s PBS supplied with 0.05% tween 20 and DMSO was used for immobilization of the protein. Recombinant human *h*TLR4/MD2 (R&D Systems, Minneapolis, MN, USA) was attached to the surface of the chip by amine coupling. The surface of flow cells 1 and 2 was initially activated with a 10-min pulse of a 1:1 mixture of 0.4 M 1-ethyl-3-(3-dimethylaminopropyl)-carbodiimide hydrochloride and 0.1 M *N*-hydroxysuccinimide, according to manufacturer recommendations. TLR4 was diluted into 10 mM sodium acetate buffer, pH 5.5, to a final concentration of 200 μg/mL, and injected twice for 600 s over the second flow cell. Both flow cells were blocked with a 10-min pulse of 1.0 M ethanolamine pH 8.5. The final capacity level was about 8000 RU. Analysis was performad in the running buffer consisting of Dulbecco’s PBS (10 mM Na_2_HPO_4_, 2 mM KH_2_PO_4_, 137 mM NaCl, 2 mM KCl, pH 7.4) supplemented with 1% DMSO (Sigma Aldrich, Darmstadt, Germany) and 1% as. All of the three hits from the cell-based assays were tested at two different concentrations: 20 μM and 200 μM. Each compound was injected for 1 min at a flow-rate of 30 μL/min, and the dissociation was monitored for 30 s. Regeneration was not needed, although 30 s of buffer-flow was used to stabilize the surface after each injection. We compared the responses obtained by the injections of the buffer and the two different sample concentrations, and continued the study with the compound that showed binding. We used the following concentrations for titration: 0, 6.25, 12.5, 25, 50, 100 and 200 μM.

### 3.7. Cytotoxicity

Standard MTT assay (Sigma Aldrich, Darmstadt, Germany) was applied according to the manufacturer’s protocol on the CHO-K1 (Chinese hamster ovary, ECACC, Salisbury, UK) in order to compare the effect of different compound within the series. The cells were cultured according to ECACC recommended conditions and seeded in a density of 8000 per well. Briefly, tested compounds were dissolved in DMSO (Sigma Aldrich, Darmstadt, Germany) and subsequently in the growth medium (F-12) so that the final concentration of DMSO did not exceed 0.5% (*v*/*v*) per well. Cells were exposed to the tested compounds for 24 h. Then the medium was replaced by a medium containing 10 μM of MTT and cells were allowed to produce formazan for another approximately 3 h under surveillance. Thereafter, medium with MTT was sucked out and crystals of formazan were dissolved in DMSO (100 µL). Cell viability was assessed spectrophotometrically by the amount of formazan produced. Absorbance was measured at 570 nm with 650 nm reference wavelength on Synergy HT (BioTek, Winooski, VT, USA). IC_50_ was then calculated from the control—subtracted triplicates using non-linear regression (four parameters) of GraphPad Prism 5 software (GraphPad Software, La Jolla, CA, USA). Final IC_50_ and SEM value was obtained as a mean of 2–3 independent measurements.

### 3.8. Ex Vivo—IL-6 Production

PBMCs was obtained by standard protocol [33] using Histopaque 1077 gradient from whole blood of healthy donors. Cells were seeded at 25,000 cells per well and incubated for 24 h with final concentration 10 µM of each compound in triplicate (*n* = 3). After 24 h 100 µL of supernatant was collected from each well and level of IL-6 was determined by human IL-6 ELISA kit obtained from Sigma Aldrich (Darmstadt, Germany). Absorbance was measured at 450 nm wavelength on Synergy HT (BioTek). Level of IL-6 was calculated from standard curve using RIA nonlinear plot in Graph Pad Prism 6.

## 4. Conclusions

This study reveals a new group of small-molecule ligands, designed by an in silico approach, that possess intrinsic agonistic activity on the *h*TLR4/MD2 and by that represent potential vaccine adjuvants. Although the observed in vitro and ex vivo immunostimulation potency is not as strong as in works of Chan et al. [17], or Jiang et al. [34], but it must be noted, that ours compounds are structurally far more distant from Lipid A and LPS and contain neither “fatty” part of LPS molecule, nor even single long lipidic chain at all. Recent thinking was that for *h*TLR4/MD2 binding at least three long acyl chains (C8–C18) is necessary to be present in the molecule to elicit the agonistic effect [9]. The direct binding of the best in vitro compound (**17**) to the *h*TLR4/MD2 receptor was confirmed by SPR study, however, this compound failed to produce IL-6 in the ex vivo study using PBMCs. On the other hand compounds **14** and **22** succeeded in this task despite of the low in vitro activity. Interestingly, high potential of **22** was suggested by in sillico studies. Therefore, further study evaluating the influence of novel compounds on Th1/Th2/Th17 bias will be needed to thoroughly assess their adjuvant and immunomodulating potential. As well as address inconsistency found among in silico, in vitro and ex vivo findings. Even though **17** did not elicit production of IL-6, compounds **14** and **22** succeeded in this task. Notably to say, all the compounds show very low cytotoxicity with IC_50_ ranging from 98 µM to 892 µM. Despite of found discrepancies we believe that by applying methods of rational drug design, we have found a novel class of TLR4 agonists which and therefore they might be regarded as novel class of potential *h*TLR4/MD2-targeting adjuvants.

## Figures and Tables

**Table 1:** Yields and melting points of compounds **14**–**25**.

Compound	R	Yield (%)	m.p. (°C)
**14**	Phenyl	81 ^a^	141.2–142.8
**15**	4-(Methyl)phenyl	92 ^a^	126.8–128.2
**16**	4-(Methoxy)phenyl	87 ^a^	132.8–134.3
**17**	4-(Chloro)phenyl	66 ^a^	150.6–152.0
**18**	3,4-(Dichloro)phenyl	75 ^a^	112.4–113.9
**19**	2-Naphtyl	92 ^a^	193.2–195.0
**20**	Cyclohexyl	59 ^a^	104.8–106.3
**21**	1-Naphtyl	80 ^a^	164.5–166.5
**22**	1,2,3,4-Tetrahydro-2-naphtyl	81 ^b^	123.2–125.2
**23**	5,6,7,8-Tetrahydro-2-naphtyl	91 ^b^	137.0–139.0
**24**	Cycloheptyl	90 ^b^	88.4–90.4
**25**	Isopentyl	87 ^b^	45.1–47.1

^a^ Yield of reaction of acyl chloride with tryptamine (Procedure A); ^b^ Yield of reaction of carboxylic acid with tryptamine (Procedure B).

**Table 2:** Mean binding energies and their standard sample deviations for **14**–**25** in *h*TRL4/MD2 (PDB ID: 4G8A) estimated by MM/PBSA method from 10 ns MD simulations.

Compound	Predicted Binding Energy (kcal/mol)	Compound	Predicted Binding Energy (kcal/mol)
**14**	−23.454 ± 5.368	**20**	−19.255 ± 4.028
**15**	−20.059 ± 6.248	**21**	−19.344 ± 6.022
**16**	−21.144 ± 4.535	**22**	−21.381 ± 4.511
**17**	−22.874 ± 3.929	**23**	−18.090 ± 3.767
**18**	−21.288 ± 3.531	**24**	−23.696 ± 2.684
**19**	−20.495 ± 4.284	**25**	−18.361 ± 2.846

**Table 3:** Cytotoxicity evaluation.

Compound	IC_50_ and SEM (μM)
**14**	297 ± 17
**15**	197 ± 41
**16**	200 ± 7
**17**	201 ± 8
**18**	892 ± 122
**19**	155 ± 12
**20**	211 ± 43
**22**	128 ± 8
**23**	98 ± 6
**24**	170 ± 10
**25**	348 ± 35

**Table 4:** Ex vivo stimulation of IL-6 production.

Compound	c (pg/mL)
**14**	53.501
**15**	-
**16**	-
**17**	-
**18**	-
**19**	-
**20**	-
**22**	147.64
LPS-RS	-
MPLA	3078.97

## Supplementary Materials

Supplementary materials are available online. Figures (S14–S25) representing overlaid conformation of each ligand (**14**–**25**) in MD study; Video 1—Record of 10 ns MD simulation of **17**.
